# The effect of cognitive behavioral therapy on depression and anxiety of women with polycystic ovary syndrome: a randomized controlled trial

**DOI:** 10.1186/s12888-023-04814-9

**Published:** 2023-05-11

**Authors:** Sheida Majidzadeh, Mojgan Mirghafourvand, Mahmoud Farvareshi, Parisa Yavarikia

**Affiliations:** 1 Department of midwifery, Student Research Committee, Faculty of Nursing and Midwifery, Tabriz University of Medical Sciences, Tabriz, Iran; 2 Social Determinants of Health Research Center, Faculty of Nursing and Midwifery, Tabriz University of Medical Sciences, Tabriz, Iran; 3 Clinical Psychologist, Razi Hospital, Tabriz University of Medical Sciences, Tabriz, Iran; 4 Department of midwifery, Faculty of Nursing and Midwifery, Tabriz University of Medical Sciences, Tabriz, Iran

**Keywords:** Polycystic ovary syndrome, Cognitive behavioral therapy, Depression, Anxiety, Quality of life

## Abstract

**Introduction:**

Women’s mental health has a significant impact on the health of society. Due to the prevalence of mental health problems in women with PCOS, this study aimed to determine the effect of cognitive behavioral therapy on depression and anxiety (primary outcomes) and quality of life (secondary outcomes) in women with polycystic ovary syndrome.

**Methods:**

This randomized controlled trial was performed on 84 patients with PCOS referred to Al-Zahra Hospital in Tabriz-Iran, 2021. Participants were randomly assigned to intervention (n = 42) and control (n = 42) groups. Counseling with cognitive behavioral therapy was provided in 8 sessions of 60–90 min weekly in groups with 5 to 7 people in each group for the intervention group. Spielberger State-Trait Anxiety, Beck Depression, and Quality of Life Questionnaire for women with polycystic ovary syndrome (PCOSQ) were used to collect data. Independent t-test and ANCOVA were used to compare the outcomes between the two groups.

**Results:**

After the intervention, based on ANCOVA test with adjusting the baseline values, mean scores of depression (mean difference (MD): -18.6; 95% confidence interval (95% CI): -19.4 to -17.8: P < 0.001), trait anxiety (MD: -15.0; 95% CI: -16.0 to -13.9; P < 0.001), and state anxiety (MD: -15.3; 95% CI: -16.2 to -14.3; P < 0.001), were significantly lower in the intervention group compared to the control group. The mean score of quality of life (MD: 19.25; 95% CI: 17.66 to 20.84; P < 0.001) was significantly higher in the intervention group compared to the control group.

**Conclusion:**

This study showed that CBT was effective in reducing depression and anxiety and improving the quality of life. Therefore it is recommended that health care providers use this approach to improve the mental health and quality of life of women with PCOS.

**Trial registration:**

Iranian Registry of Clinical Trials (IRCT): IRCT20110826007418N7. Date of registration: 25/10/2021. URL: https://en.irct.ir/trial/57348; Date of first registration: 25/10/2021.

## Background

Polycystic ovary syndrome is characterized by hyperandrogenism, polycystic ovary, oligomenorrhea, and amenorrhea. This syndrome is the most common endocrine disorder of women of reproductive age, and various factors are involved in its occurrence [1]. This disease may link to a genetic predisposition, influenced by environmental factors such as eating habits, lifestyle, and social status [2]. The prevalence of PCOS ranges between 2.2 and 26% in various countries, which can be due to different processes and methodologies in population-based studies [3]. The prevalence of this disease is between 7.1 and 14.6% in Iranian population [4]. Three criteria commonly used to evaluate the prevalence of polycystic ovary syndrome are NIH (National Institute of Health), AES (Androgen Excess Society), and Rotterdam. In some studies, PCOS is diagnosed on ultrasound modalities [5].

In PCOS, ovaries are enlarged and may contain multiple small cysts, with one or more symptoms of irregular menstruation, excessive hair growth, and infertility [6, 7]. The disease is associated with a wide range of symptoms related to reproductive and endocrine disorders. Obesity and insulin resistance are the main physiological features in patients with PCOS [8, 9]. According to studies, women with PCOS are more likely to suffer from mood disorders than healthy ones [10, 11]. Also, the prevalence of depression is higher 4–5 times in these patients [12].

Depression is one of the most important issues studied by psychologists, psychiatrists, and behavioral scientists worldwide [13]. According to the World Health Organization (WHO), depression is a common mental disorder associated with decreased mood, loss of interest, blameworthiness, worthlessness, sleep disturbance, eating problems, tiredness, and poor concentration. The prevalence of depression in women with PCOS is 37%, compared to 14.2% in healthy women [14]. PCOS is associated with inflammation; prolonged inflammation is associated with higher cortisol levels, which increases the risk of depression [15].

Anxiety is also a common mental condition today. It is an uncomfortable feeling of uneasiness and worry accompanied by physical symptoms such as shortness of breath, palpitations, high blood pressure, etc. [16]. Excessive and long-term anxiety is usually associated with physiological responses such as increased metabolism, cardiovascular function, and decreased immunity. The prevalence of anxiety symptoms is 42% in women with PCOS, compared to 8.5% in healthy women [14]. Secondary depression seems to be the most common anxiety complication [17]. Women with PCO also encounter fertility problems, leading to anticipatory anxiety about whether they can have children [18].

Relaxation, logotherapy, medication, and electroconvulsive therapy are proven to help treat people with anxiety and depression [19, 20]. Cognitive-behavioral therapy (CBT) is a combination of cognitive and behavioral approaches. CBT can help the patient recognize his distorted thoughts and ineffective behaviors. Skill acquisition and homework assignments are provided to change these thoughts and behaviors [21]. CBT is still at the forefront of psychological treatments for depressive disorders [22]. The mechanism of depression and anxiety in PCOS may be linked to the clinical features of PCOS, including obesity, insulin resistance, hyperandrogenism, inflammation, and infertility [23]. CBT is believed to repair processes that maintain depression and anxiety, such as rumination, overgeneralization, and self-focused attention [24].

So far, very little research has been done on the effect of psychological interventions, especially CBT, on depression and anxiety in women with PCOS [25, 26]; most of them have studied the prevalence and symptoms of the disease. Considering the impact of CBT on depression and anxiety and the high prevalence of mental issues in women with PCOS, we aimed to investigate the effect of this method on depression and anxiety (primary outcomes) and the quality of life (secondary outcome) of women with polycystic ovary syndrome. It was hypothesized that the CBT will alleviate depression and anxiety a well as it will improve quality of life in women with polycystic ovary syndrome.

## Methods

### Study design and participants

This randomized controlled trial with was performed in a parallel design on 84 patients with polycystic ovary syndrome referred to Al-Zahra Hospital in Tabriz-Iran, from July 2021 to October 2021.

Inclusion criteria included a minimum of secondary education, diagnosis of polycystic ovary syndrome by a gynecologist (hyperandrogenism, ovulatory dysfunction, and polycystic ovaries), and a medical record of PCOS. Exclusion criteria included having a history of mental illness according to the person’s statement, hypertension, iron deficiency anemia, diabetes, thyroid disease, epilepsy, pregnant and postpartum women.

In this study, the sample size was calculated based on both depression and anxiety variables using G-Power software. Based on the results of the study by Mehrabadi et al. (2018) [27] with considering m_1_ = 17.35 (mean score of anxiety before the intervention) and with a default reduction of 35% in anxiety score after the intervention (m_2_ = 11.27) SD_1_ = SD_2_ = 10.44, one-sided α = 0.05, power = 80%, sample size was calculated as 38 people in each group. The final sample size was 42, considering a drop-out rate of 10%. Based on the depression variable [25] with considering m_1_ = 20.35 (mean score of depression before the intervention) and with a default reduction of 35% in depression score after the intervention (m_2_ = 13.22), SD_1_ = SD_2_ = 9.82, one-sided α = 0.05, power = 90%, sample size was calculated as 34 people in each group. The final sample size was 37, considering a drop-out rate of 10%. The sample size calculated with the anxiety variable was higher; thus, the final sample size was 42 people in each group.

### Sampling

The sampling was started after the approval of the Ethics committee of Tabriz University of Medical Sciences (IR.TBZED.REC.1400.229) and registration in Iranian Registry of Clinical Trials (IRCT20110826007418N7). The researcher was referred to the gynecology & infertility clinic of Al-Zahra Hospital and given the list of women with PCOS. She contacted them and briefly explained the goals and method of the research. They were assessed based on the inclusion and exclusion criteria. Eligible and willing women were invited to attend the clinic at a specific time. The goals and methods were fully explained at the meeting then a written consent form was signed by the ones willing to participate in the study. The questionnaires of Spielberger State-Trait Anxiety Inventory (STAI) and Beck Depression Inventory (BDI) were completed. Women who scored 35–65 on STAI and above 20 on BDI were included. Then the questionnaires of socio-demographic characteristics and quality of life for women with polycystic ovary syndrome (PCOSQ) were completed. Women scored 65 or higher on STAI were referred to a psychiatrist.

### Randomization

The participants were divided into the intervention and control groups using block randomization, with 4 and 6 block sizes and an allocation ratio of 1:1; within each of these “blocks,” the conditions occurred in a random order. Random sequence generation was conducted by a person not involved in sampling and data collection. Opaque, sealed envelopes were used for allocation concealment. The envelopes were revealed sequentially as the participants entered the study.

### Intervention

The researcher conducted the group counseling in 8 sessions of 60–90 min weekly with 5–7 women in each group in a quiet room. All health and safety protocols were observed to prevent the spread of COVID-19. Online counseling was also available for whom wanted to participate due to pandemic conditions; it was provided in eight calls of 45 min weekly. Additionally, the counseling was conducted in the native language. The content of the sessions included mood screening, introducing cognitive-behavioral patterns, practicing skills, challenging thoughts, generalizing and maintaining, following up, and evaluating after the intervention. The content of the sessions was as follows:

Session 1: The researcher attempted to establish a proper relationship with the participants. She explained the number and duration of each session, group rules, problem identification, cognitive-behavioral patterns, problem description, the concept of stress and its effects, objectives, and receiving feedback from counseling sessions.

Session 2: Mood checking, presenting the progressive muscle relaxation and its practice, homework assignment: planning for progressive muscle relaxation twice a day, and receiving feedback.

Session 3: Reviewing the cognitive-behavioral pattern according to the problem, mood checking, asking the participants to explain their issues, introduction of imagery practice, homework assignment: progressive muscle relaxation, and imagery practice.

Session 4: Mood checking, reviewing the imagery practice, introduction of the first three columns of the thought record sheet (situation – automatic thoughts - emotions and mood) and the concept of hot thoughts, practicing the sheet using one of the events of last week, homework assignment: imagery practice, and receiving feedback.

Session 5: Mood checking, discussing the treatment process, reviewing the homework, introducing cognitive distortions, completing the three columns of thought record sheet (situation - automatic thoughts - emotions and mood), identification and challenging hot thoughts, homework assignment: completing the three columns of thought record sheet, and receiving feedback.

Session 6: Mood checking, reviewing the homework, presenting thought challenging and the seven-column thought record sheet (situation – automatic thoughts - emotions and mood - confirming evidence - rejecting evidence - alternative thinking - re-evaluating), completing the columns during the session, introducing the concept of challenging hot thoughts, homework assignment: relaxation techniques, and receiving feedback.

Session 7: Mood checking, reviewing the homework, completing the seven-column thought record sheet, homework assignment: completing the seven-column thought record sheet, and receiving feedback.

Session 8: Mood checking, reviewing the homework and treatment process (cognitive behavioral techniques), prevention, introducing self-management sessions, homework assignment: self-management practice.

The validity of the intervention program (CBT sessions) was reviewed and approved by the research consultant professor (third author as a psychologist) and the reviewer of the research project (another psychologist). The control group received routine drug treatments related to the disease. After the intervention, STAI, BDI, and PCOSQ questionnaires were completed again by both groups.

### Data collection tools

Socio-demographics and obstetric characteristics questionnaire, STAI, BDI, and PCOSQ were used for data collection before and after the intervention.

### Socio-demographics and obstetric characteristics questionnaire

It included questions about the age, ethnicity, marital status, childbearing, the level of education and occupation of the couples, average length of the menstrual cycle, average days of menstruation, menstrual flow volume, income sufficiency, the impact of stress on life, disease symptoms, first supporter, sexual satisfaction, infertility and evaluations in this field, duration of pregnancy attempt, PCOS duration, and the treatments used. Content and face validity were used to determine the validity of this questionnaire. It was provided to the faculty members of Tabriz University of Medical Sciences; adjustments were made based on their feedback.

### Spielberger state-trait anxiety inventory (STAI)

The concepts of state and trait anxiety were first presented by Cattell and then in further detail by Spielberger (1970). STAI has been used widely in clinical research. It includes separate self-assessment scales to measure state and trait anxiety. Scores 20–31 indicate mild anxiety, 32–42 moderate to low anxiety, 43–53 moderate anxiety, 54–64 moderately severe anxiety, and 65–75 severe anxiety. The Persian version of this tool had good validity and reliability in the research of Mehram (1373) and Panahi (1372) [28, 29].

### Beck Depression Inventory (BDI)

BDI is a self-assessment questionnaire that measures the severity of depressive symptoms. It includes 21 items, each containing four options, scored on a scale of 0 to 3. A higher score indicates the severity of the symptoms. The minimum and maximum scores are 0 and 63, respectively. BDI is designed for people aged 13 and over [30]. Scores 0–13 indicate low depression, 14–19 mild depression, 20–28 moderate depression, and 29–63 severe depression [31]. In the study of Taheri et al., psychometric properties had a high internal consistency based on Cronbach’s alpha (0.93); the correlation coefficient was calculated as 0.81 [32] .

### Quality of life questionnaire for women with polycystic ovary syndrome (PCOSQ)

PCOSQ was developed by Cronin et al. (1998) to measure the quality of life of women with PCOS. It consists of 26 items that evaluate five domains: emotions (items 2, 4, 6, 11, 14, 17, 18, 20), hirsutism (items 1, 9, 15, 16, 26), weight (items 3, 10, 12, 22, 24), infertility problems (items 5, 13, 23, 25), and menstrual problems (items 7, 8, 19, 21) [33]. The scoring is based on the 7-point Likert scale; 1: Intense / All Time − 7: No Problem / Never. A high score indicates a poorer quality of life. The Persian version of this questionnaire was presented by Amini et al. (2012); cronbach’s alpha coefficient was higher than 0.7 in the domains of emotions, hirsutism, weight, and infertility problems [33] .

In this study, the reliability of questionnaires was confirmed by testing on 20 people and determining internal consistency. Cronbach’s alpha coefficients were 0.894, 0.772, and 0.832 for STAI, BDI and PCOSQ.

### Statistical analysis

Statistical analysis was performed using SPSS version 24. The normality of the quantitative data was assessed using the Kolmogorov-Smirnov (K-S) test. Independent t tests, chi-square, chi-square by trend and Fisher’s exact tests were used to assess the homogeneity of the study groups. To compare the groups in terms of mean scores of anxiety, depression, and quality of life, independent t-test was performed before the intervention and ANCOVA test after the intervention by adjusting baseline values. All analyses were performed based on Intention-to-Treat by including all randomized patients in the analysis, regardless of what intervention they received. P < 0.05 was considered significant.

## Results

**Fig. 1:**
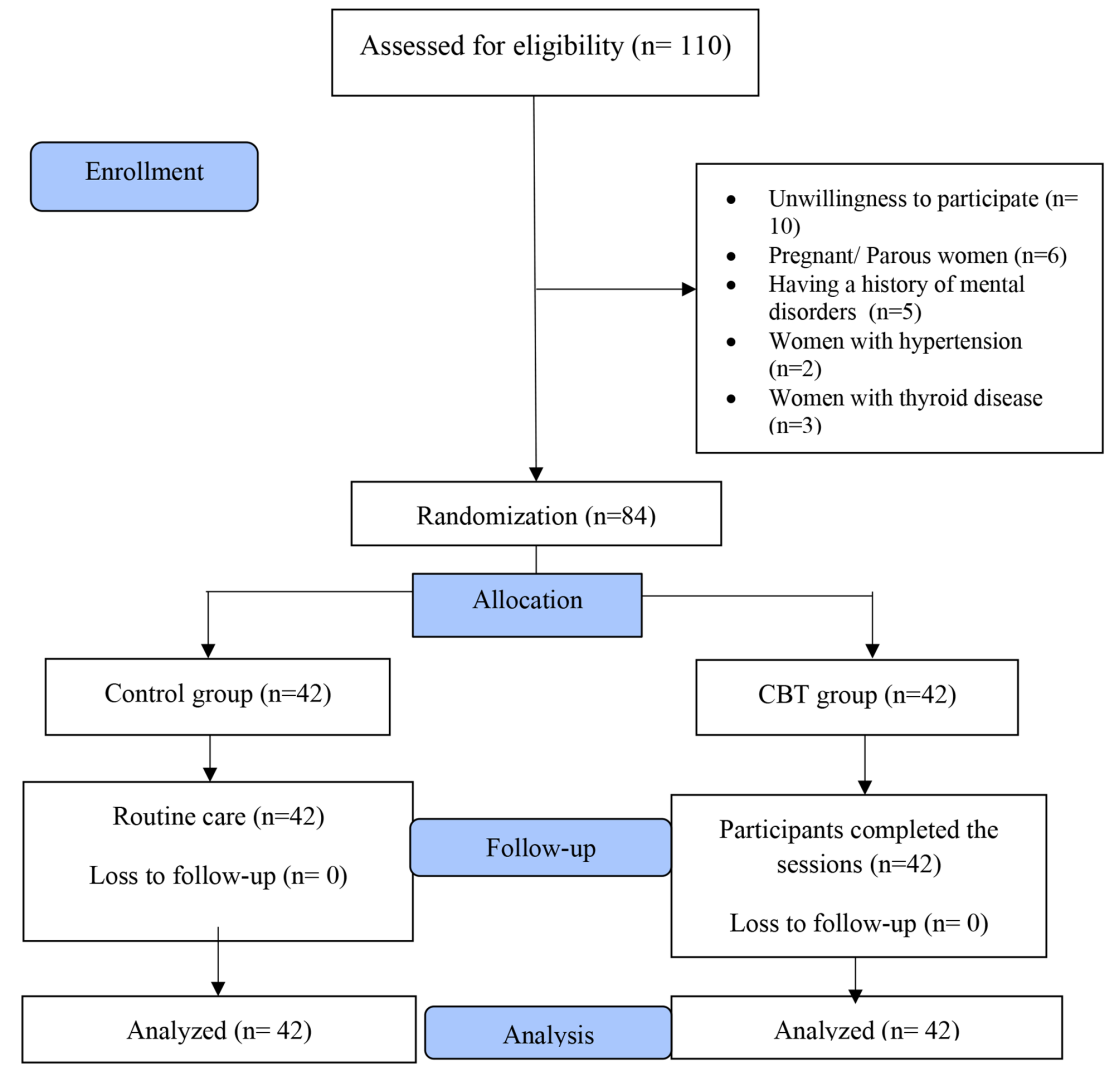
Flow chart of the study

The sampling was conducted from July through October 2021. A total of 110 women were assessed in this study. 26 women were excluded from the study due to unwillingness to participate (n = 10), having a history of childbearing (n = 6), mental illness (n = 5), hypertension (n = 2), and thyroid disease (n = 3). Among the 84 women assigned to the CBT group, all participated in 8 counseling sessions; there was no dropout from the study (Fig. 1).

The socio-demographic and obstetric characteristics of the participants are shown in Table 1. The mean (SD) age of the women was 30.3 (5.5) in the CBT group and 32.0 (4.8) in the control group. The average duration of pregnancy attempt was 5.0 (2.9) in the CBT group and 4.7 (2.5) in the control group. The average duration of the disease was 5.9 (4.8) in the CBT group and 6.8 (4.4) in the control group. The average duration of treatment was 5.0 (4.1) in the CBT group and 6.8 (4.4) in the control group. The majority of participants in both groups (97.6% in the CBT group and 97.7% in the control group) were Turk. Most of them (82.9% in the CBT group and 72.1% in the control group) were living in urban areas. All participants in the CBT group (100%) and 97.7% of the control group were married. About half of the women in the intervention group (43.9%) had a diploma; 44.2% of the control group had a university degree. Nearly half of the spouses in the control group (45.2%) had a university degree; more than a third of the intervention group (39%) had a diploma. The majority of participants in both groups (75.6% in the CBT group and 81% in the control group) had no history of childbearing. Most of them (90.2% in the CBT group and 96.8% in the control group) were housewives. Most women (92.7% in the CBT group and 83.7% in the control group) had menstrual periods of 3–7 days. The majority (75.6% in the CBT group and 83.7% in the control group) had menstrual cycles of 21–35 days. More than half of the patients in both groups (68.3% in the CBT group and 60.5% in the control group) had a moderate menstrual flow volume. More than half of them (68.3% in the CBT group and 60.5% in the control group) had somewhat sufficient income. The highest rate of complaints (53.7% in the CBT group and 72.1% in the control group) was related to infertility issues. More than half of the participants (70.7% in the CBT group and 51.2% in the control group) introduced their spouses as their first supporter. Most of them (92.6% in the CBT group and 95.2% in the control group) had moderate or complete satisfaction with their sexual lives. The majority of participants in both groups (97.6% in the CBT group and 97.6% in the control group) had infertility problems. Most of them in the intervention group (92.7%) and more than two thirds of the control group (66.7%) were not evaluated for infertility issues. There was no statistically significant difference between the groups in terms of socio-demographic and obstetric characteristics except for the couples occupation, stress role, and infertility evaluation, which was adjusted using ANCOVA (P < 0.05).

**Table 1:** Social-demographic and obstetric characteristics of participants

Characteristic	CBT^*^ (n = 42)	Control (n = 42)	P-value
	Mean (SD)	Mean (SD)	
**Age** (Year)	30.3 (5.5)	32.0 (4.8)	0.091^†^
**Duration of pregnancy attempt** (Year)	5.0 (2.9)	4.7 (2.5)	0.55^†^
**Duration of disease** (Year)	5.9 (4.8)	6.8 (4.4)	0.34^†^
**Duration of treatment** (Year)	5.0 (4.1)	6.8 (4.4)	0.53^†^
	**Number (Percent)**	**Number (Percent)**	
**Ethnicity**			1.0^¥^
Turk	40 (97.6)	42 (97.7)	
Kurd	1 (2.4)	1 (2.3)	
**Region**			0.23^‡^
City	34 (82.9)	31 (72.1)	
Town	7 (17.1)	12 (27.9)	
**Marital status**			1.0^¥^
Single	0 (0.0)	1 (2.3)	
Married	41 (100)	42 (97.7)	
**Spouse’s education**			0.37^¥^
Elementary	4 (9.8)	2 (4.8)	
Intermediate	2 (4.9)	0 (0.0)	
High school	7 (17.1)	8 (19.0)	
Diploma	16 (39.0)	13 (31.0)	
University	12 (29.3)	19 (45.2)	
**Having child**			0.55^§^
Yes	10 (24.4)	8 (19.0)	
No	31 (75.6)	34 (81.0)	
**Spouse’s job**			0.003^¥^
Unemployed	2 (4.9)	0 (0.0)	
Worker	18 (43.9)	9 (21.4)	
Employee	6 (14.6)	10 (23.8)	
Shopkeeper	3 (7.3)	15 (35.7)	
Retired	0 (0.0)	0 (0.0)	
Other	12 (29.3)	8 (19.0)	
**Woman job**			0.02^§^
House wife	37 (90.2)	30 (96.8)	
Employed	4 (9.8)	13 (30.2)	
**Woman education**			0.19^‡^
Intermediate	7 (17.1)	4 (9.3)	
High school	6 (14.6)	9 (20.9)	
Diploma	18 (43.9)	11 (25.6)	
University	10 (24.4)	19 (44.2)	
**Days of menstruation**			0.48^¥^
< 3 days	1 (2.4)	4 (9.3)	
3–7 days	38 (92.7)	36 (83.7)	
> 7 days	2 (4.9)	3 (7.0)	
**Length of the menstrual cycle**			0.26^¥^
< 21 days	5 (12.2)	1 (2.3)	
21–35 days	31 (75.6)	36 (83.7)	
> 35 days	5 (12.2)	6 (14.0)	
**Menstrual flow volume**			0.72^¥^
Light	4 (9.8)	7 (16.3)	
Moderate	28 (68.3)	26 (60.5)	
Heavy	9 (22.0)	9 (20.9)	
Very heavy	0 (0.0)	1 (2.3)	
**Income sufficiency**			0.10^¥^
Not sufficient	12 (29.3)	10 (23.3)	
Somewhat sufficient	28 (68.3)	26 (60.5)	
Sufficient	1 (2.4)	7 (16.3)	
**Stress Role**			0.003^§^
Low	8 (19.5)	2 (4.7)	
Moderate	15 (36.6)	31 (72.1)	
High	18 (43.9)	10 (23.3)	
**Disease symptoms**			0.21^¥^
Obesity	2 (4.9)	4 (9.3)	
Hirsutism	3 (7.3)	2 (4.7)	
Acne	3 (7.3)	2 (4.7)	
Menstrual problems	11 (26.8)	4 (9.3)	
Infertility problems	22 (53.7)	31 (72.1)	
**First supporter**			0.18^¥^
Mother	5 (12.2)	10 (23.3)	
Father	1 (2.4)	1 (2.3)	
Mother & Father	5 (12.2)	10 (23.3)	
Spouse	29 (70.7)	22 (51.2)	
Health personnel	1 (2.4)	0 (0.0)	
**Sexual satisfaction**			0.11^¥^
Dissatisfied	0 (0.0)	0 (0.0)	
Rarely satisfied	3 (7.3)	2 (4.8)	
Somewhat satisfied	19 (46.3)	29 (69.0)	
Satisfied	19 (46.3)	11 (26.2)	
**Infertility problems**			1.0^¥^
Yes	40 (97.6)	41 (97.6)	
No	1 (2.4)	1 (2.4)	
**Infertility evaluation**			0.003^§^
Yes	3 (7.3)	14 (33.3)	
No	38 (92.7)	28 (66.7)	

^*^ Cognitive Behavioral Therapy; ^†^Independent t-test; ^‡^Chi-square for trend; ^§^Chi-square; ^¥^ Fisher’s exact test

The mean (SD) depression score was 24.4 (5.0) before the intervention in the CBT group which decreased to 6.7 (4.3) after the intervention. It was 23.3 (3.2) and 24.5 (2.8) in the control group before and after the intervention, respectively. Based on the independent t-test, there was no statistically significant difference between the groups before the intervention (P = 0.25). Based on the ANCOVA test with adjusting the baseline scores, the mean depression score in the CBT group was significantly less than the control one after the intervention (MD: -18.6; 95% CI: -19.4 to -17.8; P < 0.001) (Table 2).

**Table 2:** Comparison of the mean score of depression and anxiety among study groups

Variable	CBT^*^ (n = 42) Mean (SD^†^)	Control (n = 42) Mean (SD^†^)	Mean Difference (95% Confidence Interval)	P-value
**Depression** (Score range: 0 to 63)				
Before intervention	24.4 (5.0)	23.3 (3.2)	-1.0 (2.9 to 0.7)	0.25^‡^
After intervention	6.7 (4.3)	24.5 (2.8)	-18.6 (-19.4 to -17.8)	< 0.001^§^
**State anxiety** (Score range: 20 to 80)				
Before intervention	42.9 (2.9)	42.2 (2.9)	0.7 (-2.0 to 0.5)	0.27^‡^
After intervention	29.0 (3.9)	42.3 (2.8)	-15.3 (-16.2 to -14.3)	< 0.001^§^
**Trait anxiety** (Score range: 20 to 80)				
Before intervention	41.8 (2.8)	41.1 (2.9)	0.7 (-1.9 to 0.5)	0.25^‡^
After intervention	29.3 (4.3)	41.3 (2.9)	-15.0 (-16.0 to -13.9)	< 0.001^§^

^*^ Cognitive Behavioral Therapy; ^†^ Standard Deviation; ^‡^Independent t-test; ^§^ ANCOVA with adjusting the baseline value and the variables of couples occupation, stress role, and infertility evaluation

The mean (SD) state anxiety score was 42.9 (2.9) before the intervention in the CBT group which decreased to 29.0 (3.9) after the intervention. It was 42.2 (2.9) and 42.3 (2.8) in the control group before and after the intervention, respectively. Based on the independent t-test, there was no statistically significant difference between the groups before the intervention (P = 0.27). According to the ANCOVA test and adjusting the baseline scores, the mean state anxiety score in the CBT group was significantly less than the control one after the intervention (MD: -15.3; 95% CI: -16.2 to -14.3; P < 0.001) (Table 2).

The mean (SD) trait anxiety score was 41.8 (2.8) before the intervention in the CBT group which decreased to 29.3 (4.3) after the intervention. It was 41.1 (2.9) and 41.3 (2.9) in the control group before and after the intervention, respectively. Based on the independent t-test, there was no statistically significant difference between the groups before the intervention (P = 0.25). According to the ANCOVA and adjusting the baseline scores, the mean trait anxiety score in the CBT group was significantly less than the control one after the intervention (MD: -15.0; 95% CI: -16.0 to -13.9; P < 0.001) (Table 2).

The mean (SD) quality of life score was 70.2 (13.3) before the intervention in the CBT group which increased to 89.0 (12.9) after the intervention. It was 71.9 (14.5) and 71.4 (4.1) in the control group before and after the intervention, respectively. Based on the independent t-test, there was no statistically significant difference between the groups before the intervention (P = 0.57). According to the ANCOVA with adjusting the baseline scores, the mean quality of life score in the CBT group was significantly more than the control one after the intervention (MD: 19.2; 95% CI: 17.6 to 20.8; P < 0.001). There was no statistically significant difference between the groups before the intervention in terms of quality of life domains. The mean score of all domains except hirsutism in the CBT group was significantly more than the control group after the intervention (Table 3).

**Table 3:** Comparison of the mean scores of total quality of life and its subscales among study groups

Variable	CBT^*^ (n = 42) Mean (SD^†^)	Control (n = 42) Mean (SD^†^)	Mean Difference (95% Confidence Interval)	P-value
**Hirsutism** (Score range: 5 to 35)				
Before intervention	14.8 (4.9)	15.3 (6.1)	0.5 (1.8 to 3.0)	0.63^‡^
After intervention	14.5 (4.6)	14.8 (6.1)	0.3 (-0.5 to 0.9)	0.61^§^
**Infertility problems** (Score range: 4 to 28)				
Before intervention	8.4 (3.4)	9.8 (3.6)	1.4 (1.0 to 2.9)	0.06^‡^
After intervention	12.1 (2.8)	9.4 (3.6)	3.8 (3.0 to 4.6)	< 0.001^§^
**Weight** (Score range: 5 to 35)				
Before intervention	14.8 (3.9)	13.7 (4.5)	1.0 (2.9 to 0.7)	0.24^‡^
After intervention	19.7 (3.9)	14.3 (4.2)	4.5 (3.5 to 5.4)	< 0.001^§^
**Emotions** (Score range: 8 to 56)				
Before intervention	21.9 (4.6)	22.6 (5.3)	0.7 (1.4 to 2.8)	0.51^‡^
After intervention	29.2 (5.0)	22.7 (4.7)	7.1 (6.2 to 8.0)	< 0.001^§^
**Menstrual problems** (Score range: 4 to 28)				
Before intervention	10.1 (2.3)	10.2 (2.6)	0.03 (1.0 to 1.1)	0.94^‡^
After intervention	13.3 (2.5)	10.0 (2.5)	3.3 (2.9 to 3.7)	< 0.001^§^
**Total Quality of life** (Score range: 26 to 182)				
Before intervention	70.2 (13.3)	71.9 (14.5)	1.7 (-4.3 to 7.7)	0.57^‡^
After intervention	89.0 (12.9)	71.4 (4.1)	19.2 (17.6 to 20.8)	< 0.001^§^

^*^ Cognitive Behavioral Therapy; ^†^ Standard Deviation; ^‡^Independent t-test; ^§^ ANCOVA with adjusting the baseline value and the variables of couples occupation, stress role, and infertility evaluation

## Discussion

The results showed that CBT was effective in reducing depression and anxiety and promoting the quality of life of women with the PCOS.

CBT significantly reduced the mean depression score in this study. In a research by Rofey et al. (2009), women with PCOS, depression, and obesity participated in eight weekly CBT sessions focusing on lifestyle changes, medical history, and psychological education. The SECA (a calibrated weighing scale) and CDI (Children’s Depression Inventory) scales were used to measure changes in weight and depression. Follow-ups showed a significant decrease in mean weight and depression score after the intervention [34]. Correa et al. (2015) investigated the effect of the cognitive-behavioral approach on patients with PCOS. Beck Depression Inventory-II (BDI-II) questionnaire was used to measure the rate of depression before and after the intervention. The counseling sessions focused on teaching physiological and psychological symptoms, thought challenges, stress and anxiety management strategies, and self-concept. The mean score of depression was significantly reduced after the intervention; improvement of general function were also reported [35]. The results of both studies are in line with the present one. According to recent studies, the CBT approach can provide better improvements in depression than other approaches by affecting repetitive negative thinking (RNT) and focusing on reducing these thoughts [36]. It restructures negative thought patterns, corrects misconceptions, changes attentional direction, and creates adaptive coping thought patterns [37].

CBT significantly reduced the mean anxiety score in this study. Nobakht et al. (2018) investigated the effect of cognitive-behavioral counseling on anxiety in women with HIV. Six weekly counseling sessions were conducted based on a review of mood, awareness, self-image, and experiences, along with questions about the desire to change lifestyles, stress reduction strategies, mental focus, and relaxation. The Depression, Anxiety and Stress Scale (DASS-21) was used to measure anxiety. The results showed a significant reduction in anxiety scores and improvements in patient mood [38]. In a review study by Golshani et al. (2020), the results showed a significant reduction in the mean anxiety score in the intervention group with CBT in pregnant women with infertility history [39]. The results of both studies are consistent with the present one. CBT aims to stop negative cycles by breaking down issues that make the individual anxious or scared. Making concerns more manageable, CBT can help change negative patterns and improve feelings [40].

In this study, CBT significantly improved the mean score of quality of life in domains of menstrual problems, weight, infertility, and emotional problems. Cooney et al. (2018) studied the effect of CBT on obese women with PCOS and depressive symptoms. The counseling was provided in 8 sessions of 30 min weekly. PCOSQ scale was used to measure the quality of life before and after the intervention. The content of the sessions was based on planning and practicing cognitive skills such as identifying automatic thoughts and cognitive distortions. The participants in the counseling group reported a significant decrease in weight; the quality of life score was increased compared to the lifestyle change group [41]. Abdollahi et al. (2019) investigated the effect of cognitive-behavioral counseling on the quality of life of women with PCOS. The PCOSQ scale was used to measure the quality of life of study groups. The content of the meetings included cognitive-behavioral patterns, self-concept, breathing technique and timing, nutrition, lifestyle, muscle relaxation, stress management, and positive expression. After the intervention, the mean quality of life score in the counseling group increased significantly compared to the control group [25]. Another study by Jalilian et al. (2018) aimed to determine the effect of cognitive-behavioral counseling on the quality of life of women with PCOS. The PCOSQ scale was used to measure the quality of life in both groups. Counseling with CBT approach significantly improved the mean score of quality of life in hirsutism, weight, infertility, and emotional problems other than menstrual problems [42]. The results of all studies are consistent with the present one. CBT uses practical self-help strategies, designed to improve the quality of life. It focuses on increasing awareness, challenging thoughts, stress management skills, increasing hope, relaxation, and empowering women [43].

In recent years, psychological problems caused by PCOS has been drawn much attention from researchers worldwide. In addition to being a fertility and beauty issue for women, it can lead to psychological disorders such as depression and anxiety [44]. Physical symptoms caused by PCOS are one of the causes of psychological disorders in these women; they are three times more prone to psychological disorders and have a lower quality of life [45]. CBT approach can reduce depression and anxiety and improve the quality of life of women with PCOS using a combination of methods such as cognitive assessment techniques, identification of distortions, and coping skills [46].

## Strengths and limitations

Observing all the principles of clinical trials, including random allocation and allocation concealment, was among the strengths of our study. Content design and consulting intervention were based on the cultural and moral values ​​of the region. There was no drop-out from the study; all the participants were analyzed. Due to the nature of the intervention, blinding the participants, researcher, and outcome assessor was impossible.

## Conclusion

In this study, CBT reduced the mean scores of anxiety and depression and improved the quality of life of women with PCOS. Due to the prevalence of mental disorders in these women, we recommend that health care providers develop programs based on CBT to promote the mental health and quality of life of women with PCOS.

## Data Availability

The datasets generated and/or analyzed during the current study are not publicly available due to limitations of ethical approval involving the patient data and anonymity but are available from the corresponding author on reasonable request.

## Abbreviations

CBT: Cognitive-Behavioral Therapy
ANCOVA: Analysis of Covariance
95% CI: 95% confidence interval
IRCT: Iranian Registry of Clinical Trials
STAI: Spielberger State-Trait Anxiety Inventory
BDI: Beck Depression Inventory
PCOSQ: Quality of Life Questionnaire for women with polycystic ovary syndrome
SD: standard deviation
